# Determining the optimal fasting glucose target for patients with type 2 diabetes: Results of the multicentre, open‐label, randomized‐controlled FPG GOAL trial

**DOI:** 10.1111/dom.13733

**Published:** 2019-06-10

**Authors:** Wenying Yang, Jianhua Ma, Guoyue Yuan, Ling Li, Min Zhang, Yibing Lu, Xinhua Ye, Weihong Song, Ming Liu, Jun Wu, Riqiu Chen, Yunguang Li, Xia Zhang, Nan Cui, Jinkui Yang

**Affiliations:** ^1^ Endocrine Department China‐Japan Friendship Hospital Beijing China; ^2^ Endocrine Department, Nanjing First Hospital Nanjing Medical University Nanjing China; ^3^ Endocrine Department Affiliated Hospital of Jiangsu University Zhenjiang China; ^4^ Endocrine Department Shengjing Hospital of China Medical University Shenyang China; ^5^ Endocrine Department, Qingpu Branch of Zhongshan Hospital Affiliated with Fudan University Shanghai China; ^6^ Endocrine Department, The Second Affiliated Hospital Nanjing Medical University Nanjing China; ^7^ Endocrine Department, Changzhou Second People's Hospital Affiliated with Nanjing Medical University Changzhou China; ^8^ Endocrine Department Chenzhou No. 1 People's Hospital Chenzhou China; ^9^ Endocrine Department Tianjin Medical University General Hospital Tianjin China; ^10^ Endocrine Department The Third Hospital of Wuhan Wuhan China; ^11^ Endocrine Department Lishui People's Hospital Lishui China; ^12^ Medical Department Sanofi Investment Co., Ltd. Shanghai China; ^13^ Endocrine Department Beijing Tongren Hospital Beijing China

**Keywords:** fasting blood glucose, glycated haemoglobin, insulin glargine, type 2 diabetes

## Abstract

The optimal fasting blood glucose (FBG) target of achieving HbA1c less than 7.0% in type 2 diabetes (T2D) patients remains controversial. This open‐label trial randomized (1:3:3) 947 adults with uncontrolled T2D (HbA1c >7% to ≤10.5%) who were using one to three oral antidiabetic drugs to achieve an FBG target of 3.9 < FBG ≤5.6 mmol/L (Group 1), 3.9 < FBG ≤6.1 mmol/L (Group 2) or of 3.9 < FBG ≤7.0 mmol/L (Group 3). Targets were achieved using a pre‐defined insulin glargine 100 U/mL titration scheme. The primary endpoint was proportion of patients achieving HbA1c <7.0% at 24 weeks. At 24 weeks, 44.4%, 46.1% and 37.7% of patients achieved HbA1c <7.0% in Groups 1, 2 and 3, respectively (*P* = 0.017; Group 2 vs Group 3). Alert hypoglycaemia (glucose ≤3.9 mmol/L) was significantly more frequent in Group 1 than in Group 3 (38.9 vs 23.3%; *P* < 0.001) but was not in Group 2 vs Group 3 (27.5% vs 23.3%; *P* = 0.177). Clinically important hypoglycaemia (glucose ≤3.0 mmol/L) was reported in 4.8%, 2.0% and 3.8% of patients in Groups 1, 2 and 3, respectively. In conclusion, the optimal FBG target for most Chinese patients with T2D appears to be 3.9‐6.1 mmol/L.

## 1 INTRODUCTION

Most current guidelines for management of type 2 diabetes (T2D) recommend that patients maintain glycated haemoglobin (HbA1c) levels below 7% to achieve long‐term glucose control.^1^, ^2^, ^3^, ^4^, ^5^ While a goal of HbA1c <7.0% is given in guidelines as suitable for the majority of patients with T2D,^1^, ^2^, ^3^, ^4^, ^5^ the corresponding fasting blood glucose (FBG) target to achieve this goal is not clearly defined. Guidelines from the American Diabetes Association (ADA) in 2018 recommend an FBG target of 4.4‐7.2 mmol/L to achieve HbA1c <7%.^1^ Guidelines from the American Association of Clinical Endocrinologists‐American College of Endocrinology in 2018 and from the International Diabetes Federation in 2017 recommend an FBG target of <6.1 mmol/L to achieve HbA1c <6.5%^3^ and < 7%,^4^ respectively. Thus, the optimal FBG target to achieve HbA1c <7.0% in patients with T2D remains controversial.

Only one study, conducted in a Western country, demonstrated that more patients randomized to an FBG target of 3.9‐5.0 mmol/L achieved HbA1c <7.0% than those randomized to an FBG target of 4.4‐6.1 mmol/L.^6^ However, the proportion of patients who achieved HbA1c <7% without hypoglycaemia (glucose ≤3.1 mmol/L) was the same in the two target groups.^6^ The present study was designed to evaluate the effect of three pre‐defined FBG targets on the proportion of Chinese patients with T2D who achieved HbA1c <7.0%.^7^ The FBG targets to which patients were randomized were 3.9 < FBG ≤7.0, 3.9 < FBG ≤6.1 and 3.9 < FBG ≤5.6 mmol/L. Titration with basal insulin glargine 100 U/mL was chosen to provide FBG control. In summary, this study aimed to elucidate the optimal FBG target for patients with T2D to maximize the proportion of patients to achieve HbA1c <7% while minimizing hypoglycaemia risk.

## 2 MATERIALS AND METHODS

### 2.1 Participants

This was a 24 week, open‐label, parallel‐group, randomized, treat‐to‐target study (ClinicalTrials.gov identifier: NCT02545842). The study design and methods have been reported previously.^7^ Briefly, eligible patients were between 18 and 65 years of age, had a diagnosis of T2D, with HbA1c >7% to ≤10.5% despite stable doses of one to three oral anti‐hyperglycaemic drugs (OADs) for at least 3 months, had fasting plasma glucose (FPG) of >7 mmol/L, had a body mass index of ≥20 to ≤40 kg/m^2^, had a duration of diabetes of at least 1 year, and were willing to initiate treatment with basal insulin. Patients were randomly assigned (1:3:3) to one of three FBG target groups: 3.9 < FBG ≤ 5.6 mmol/L (Group 1), 3.9 < FBG ≤ 6.1 mmol/L (Group 2) or 3.9 < FBG ≤ 7.0 mmol/L (Group 3). Randomization was stratified by use of sulfonylurea.

The study was conducted in accordance with the principles of the Declaration of Helsinki and in line with the International Conference on Harmonisation guidelines for good clinical practice. An institutional review board at each site approved the study, and all participants gave written informed consent.

### 2.2 Procedures

At randomization, all study patients initiated subcutaneous once‐daily insulin glargine 100 U/mL (Lantus SoloSTAR, Sanofi‐Aventis Deutschland GmbH, Frankfurt, Germany) at a dose of 0.2 U/kg. During the study, patients measured FBG daily, using a blood glucose meter, and study physicians reviewed the self‐monitored FBG values and titrated the basal insulin dose at each study visit (Table S1). The lowest value of the last three consecutive self‐monitored FBG values was used for decisions concerning insulin titration at each visit.

Patients continued to receive baseline OADs for the duration of the study; these could be decreased or discontinued only at the investigators' discretion based on safety and in accordance with Chinese treatment guidelines and local label indications.

### 2.3 Endpoints and assessments

The primary endpoint was the proportion of patients achieving HbA1c <7% at 24 weeks. Secondary endpoints included the proportion of patients achieving HbA1c <7% without hypoglycaemia (blood glucose ≤3.9 [alert] or ≤ 3.0 mmol/L [clinically important]) at 24 weeks, change from baseline in HbA1c, FBG, postprandial glucose and FPG at 24 weeks, distribution of patients with FPG or FBG ≤5.6 mmol/L, 5.6‐6.1 mmol/L, 6.1‐7.0 mmol/L and > 7.0 mmol/L at Week 24, final insulin dose in each arm at the end of the study, frequency and incidence of hypoglycaemia (definitions provided in Table S2) and changes in body weight. Exploratory endpoints included the proportion of patients with HbA1c <7% according to groups re‐divided by actual 24‐week FPG levels, and the relationship between mean FBG over one to 12 weeks and HbA1c at 12 weeks and between mean FBG over 13 to 24 weeks and HbA1c at 24 weeks. Additional safety outcomes included frequency and severity of adverse events (AEs) were coded using MedDRA version 18.1. Sample size calculation and methods of statistical analysis are provided online in Supporting Information.

## 3 RESULTS

### 3.1 Participants

Between 7 September 2015 and 20 April 2018, 947 patients from 44 sites in China were randomly assigned to Group 1 (n = 136), Group 2 (n = 405) or Group 3 (n = 06). Of these, 885 patients completed the study. The most common reasons for study discontinuation were patient withdrawal and protocol violations (Figure S1). Demographic and baseline characteristics were similar across groups (Table 1). Patients had a mean age of 53.9 years (56.2% male) and a mean duration of diabetes of 7.9 years. The majority of patients were receiving two OADs at baseline (64.5%) and 62.3% were receiving treatment with sulphonylureas.

**Table 1:** Baseline characteristics in the full analysis set

Characteristic	FBG target > 3.9 mmol/L to			Total (n = 914)	*P* value^a^
	≤5.6 mmol/L (n = 126)	≤6.1 mmol/L (n = 393)	≤7.0 mmol/L (n = 395)		
Age, y	54.1 ± 7.2	54.2 ± 7.4	53.5 ± 7.4	53.9 ± 7.4	0.392^b^
Male, n (%)	69 (54.8)	214 (54.5)	231 (58.5)	514 (56.2)	0.490^c^
Disease duration, y	8.2 ± 5.5	8.0 ± 4.7	7.8 ± 4.8	7.9 ± 4.8	0.707^b^
HbA1c, %	8.50 ± 0.91	8.63 ± 0.92	8.57 ± 0.94	8.59 ± 0.92	0.314^b^
HbA1c, mmol/mol	69 ± 9.9	71 ± 10.1	70 ± 10.3	70 ± 10.1	0.314^b^
Serum FPG, mmol/L	10.4 ± 2.2	10.6 ± 2.2	10.5 ± 2.3	10.5 ± 2.3	0.636^b^
Self‐monitored FBG, mmol/L	9.3 ± 1.9	9.6 ± 2.0	9.5 ± 2.0	9.5 ± 2.0	0.287^b^
PPG, mmol/L	13.2 ± 3.8	13.8 ± 3.7	13.9 ± 3.7	13.8 ± 3.7	0.226^b^
Insulin dose, U/d	12.2 ± 2.5	12.7 ± 2.7	12.6 ± 2.8	12.6 ± 2.7	0.284^b^
Insulin dose, U/kg.d	0.18 ± 0.03	0.18 ± 0.03	0.18 ± 0.03	0.18 ± 0.03	0.417^b^
Weight, kg	69.6 ± 11.6	70.5 ± 11.7	70.1 ± 11.2	70.2 ± 11.4	0.757^b^
BMI, kg/m^2^	25.5 ± 3.0	25.6 ± 3.0	25.6 ± 3.2	25.6 ± 3.1	0.936^b^
Sulphonylurea use, n (%)	82 (65.1)	243 (61.8)	244 (61.8)	569 (62.3)	0.780^c^
Glimepiride	46 (36.5)	114 (29.0)	121 (30.6)	281 (30.7)	
Gliclazide	21 (16.7)	92 (23.4)	86 (21.8)	199 (21.7)	
Other	15 (11.9)	37 (9.4)	37 (9.4)	89 (9.7)	
Number of OADs, n (%)					0.408^d^
1	18 (14.3)	65 (16.6)	52 (13.2)	135 (14.8)	
2	84 (66.7)	253 (64.7)	251 (63.5)	588 (64.5)	
3	24 (19.0)	73 (18.7)	92 (23.3)	189 (20.7)	

*Note*: Values are presented as mean ± standard deviation unless otherwise stated.

Abbreviations: BMI, body mass index; FBG, fasting blood glucose; FPG, fasting plasma glucose; OADs, oral anti‐hyperglycaemic drugs; PPG, postprandial glucose.

a Global nominal p‐values assessing differences between the three FBG target groups.

b ANOVA.

c Fisher's Exact Test.

d Chi‐Squared Test.

### 3.2 Efficacy

#### 3.2.1 Primary endpoint

**Figure 1:**
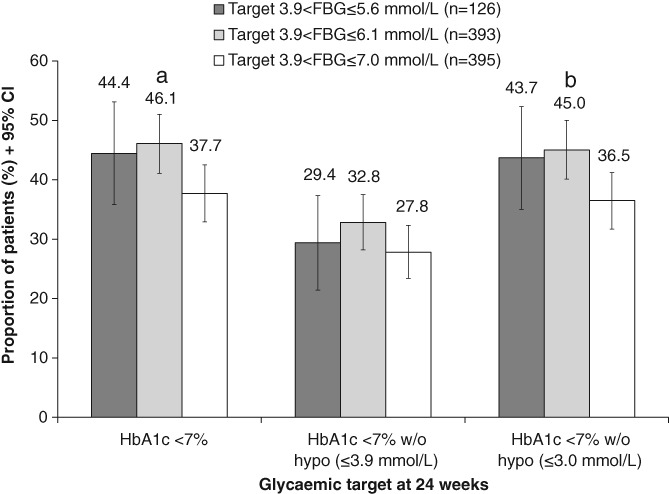
Achievement of HbA1c < 7% and HbA1c < 7% without hypoglycaemia at 24 weeks by FBG target group (n = 914, LOCF, FAS). Abbreviations: FAS, full analysis set; FBG, fasting blood glucose; hypo, hypoglycaemia; LOCF, last observation carried forward; w/o, without. ^a^ *P* < 0.025 for Bonferroni‐adjusted analysis; *nominal *P* < 0.05 vs FBG ≤7.0 mmol/L

At 24 weeks, 44.4%, 46.1% and 37.7% of patients had an HbA1c level less than 7% in Groups 1, 2 and 3, respectively (Figure 1). The proportion of patients achieving HbA1c <7% was numerically, but not significantly, higher in Group 1 vs Group 3 (*P* = 0.183); thus, the second fixed‐sequence hypothesis test was not performed. E xploratory Bonferroni‐adjusted (α < 0.025) analysis showed a significant difference in the proportion of patients achieving HbA1c <7% in Group 2 vs Group 3 (*P* = 0.017).

#### 3.2.2 Secondary endpoints

No difference was observed among groups in the proportion of patients who achieved HbA1c <7% without alert hypoglycaemia (≤3.9 mmol/L) (Figure 1). In contrast, a significantly greater proportion of patients in Group 2 achieved HbA1c <7%, without clinically important hypoglycaemia (≤3.0 mmol/L), than in Group 3 (45.0% vs 36.5% of patients; *P* = 0.014) (Figure 1).

All patients experienced mean reductions from baseline in glycaemic parameters (Table S3). However, patients in Groups 1 and 2 experienced significantly greater reductions from baseline in HbA1c, FBG and FPG at 24 weeks as compared to patients in Group 3 (Table S3).

After 24 weeks, 70.1%, 67.6% and 79.0% of patients in Groups 1, 2 and 3, respectively, had FBG values within their pre‐defined target range according to a pre‐planned titration strategy based on lowest value of the last three consecutive FBG values. Detailed distribution of FBG and FPG in each group is described in Table S4.

The doses of insulin administered at 24 weeks to patients in Groups 1 and 2 were significantly greater (*P* < 0.001) than the dose administered to patients in Group 3 (Table S3).

#### 3.2.3 Exploratory endpoints

The proportion of patients with HbA1c less than 7%, according to groups re‐divided by actual 24‐week FPG levels, and the relationship between mean FBG and HbA1c are described online.

### 3.3 Safety

Overall, 65% of patients experienced an AE during the 24‐week treatment period, the majority of which were mild in severity. The most common AEs were hypoglycaemia, upper respiratory tract infections, nasopharyngitis and toothache (Table S5). Less than 2% of the study population discontinued the study because of an AE and no deaths occurred during the treatment period.

Alert hypoglycaemia (≤3.9 mmol/L) was significantly more frequent in Group 1 than in Group 3 (38.9 vs 23.3%; *P* < 0.001) but was not significantly more frequent in Group 2 than in Group 3 (27.5% vs 23.3%; *P* = 0.177). Similar findings were seen concerning any hypoglycaemia, symptomatic hypoglycaemia and nocturnal hypoglycaemia. However, there were no significant differences in clinically important hypoglycaemia (≤3.0 mmol/L) among the three groups (4.8%, 2.0% and 3.8%, respectively; all *P* ≥ 0.05). Severe hypoglycaemia occurred in only one patient each in Groups 2 and 3 (Table S5). The incidence rate of hypoglycaemia was similar to the frequency of hypoglycaemia, with the exception of the “any hypoglycaemia” category (Table S5).

Although body weight increased by 0.5‐0.7 kg in all target FBG groups, no significant between‐group differences in change from baseline in body weight were observed (Table S5).

## 4 DISCUSSION

To our knowledge, this is the first prospective randomized study to examine the effect of three FBG targets on HbA1c control among patients with T2D, affording the opportunity to identify an optimal FBG target associated with a balanced efficacy and safety profile. The results showed that the proportion of patients who achieved HbA1c <7% or HbA1c <7% without clinically important hypoglycaemia (≤3.0 mmol/L) was significantly higher in the 3.9 < FBG ≤6.1 mmol/L target group than in the 3.9 < FBG ≤7.0 mmol/L target group (46.1% vs 37.7% or 45.0% vs 36.5%). The frequency or incidence rate of hypoglycaemia did not differ significantly between patients randomized to the 3.9 < FBG ≤6.1 mmol/L target and those randomized to the 3.9 < FBG ≤7.0 mmol/L target, with the exception of the incidence rate of the “any hypoglycaemia” category. Taken together, these findings suggest that a 3.9 < FBG ≤6.1 mmol/L target represents the optimal balance between glycaemic control and safety.

One previously published study evaluated the effect of two different FBG targets (3.9‐5.0 mmol/L or 4.4‐6.1 mmol/L) on HbA1c control; however, it had some limitations.^6^ Although more patients achieved HbA1c <7.0% with the lower FBG target (64.3% vs 54.5%; *P* = 0.04), the proportion of patients achieving HbA1c <7.0% without hypoglycaemia (≤3.0 mmol/L) was the same (44.6% vs 44.6%; *P* = 0.796).^6^ The study examined only two FBG targets, both weighted towards the lower end of the FBG spectrum, limiting its ability to reveal an FBG target with an optimal efficacy and safety profile compared with a higher FBG target, such as the 7.0 mmol/L target recommended by the ADA and by Chinese guidelines.^1^, ^5^ In contrast, our study, which employed three FBG targets, was better able to identify a 3.9 < FBG ≤6.1 mmol/L target as representative of the optimal balance between efficacy and safety for the majority patients with T2D.

This study has limitations. While the 3.9 < FBG ≤5.6 mmol/L target group experienced a significantly larger reduction in HbA1c at 24 weeks, as compared with the 3.9 < FBG ≤7.0 mmol/L target group, there was no significant difference in the proportion of patients who achieved HbA1c <7% between these target groups. This is possibly explained by the fact that our sample size calculations assumed a difference of 15% in achieving HbA1c <7% between the 3.9 < FBG≤5.6 mmol/L and 3.9 < FBG≤7.0 mmol/L target groups.^8^ However, the actual between‐group difference was approximately7%; thus, the study was underpowered to detect these differences.

In conclusion, our study showed a balanced efficacy and safety profile among patients with an FBG target of 3.9 to 6.1 mmol/L, suggesting that this could be an optimal target for the majority of patients with T2D.

## CONFLICT OF INTEREST

W. Y. has received honoraria for speakers' bureau and advisory board participation from Sanofi Aventis, Novo Nordisk, AstraZeneca, Bayer, Boehringer Ingelheim, Eli Lily, Merck Sharp & Dohme, Merck and Servier; and has also received investigator‐initiated trial research grants from AstraZeneca, outside of the submitted work. L. L. has received honoraria for speakers' bureau participation from Sanofi Aventis, Novo Nordisk, Bayer, Takeda, Eli Lily, Merck and Gan & Lee, outside of the submitted work. R. C. has received honoraria for speakers' bureau participation from Sanofi Aventis and Novo Nordisk, outside of the submitted work. X. Y. has received honoraria for speakers' bureau participation from AstraZeneca, Merck Sharp & Dohme and Novo Nordisk, outside of the submitted work. J. M., G. Y., M. Z., Y. L., W. S., M. L., J. W., J. Y. have no conflict of interests to disclose. Y. L., X. Z. and N. C. are employees of Sanofi China.

## AUTHOR CONTRIBUTIONS

The study was designed by W. Y. W. Y., J. M., G. Y., L. L., M. Z., Y. L., X. Y., W. S., M. L., J. W., R. C. and J. Y. were in charge of individual study centres; they recruited patients and conducted the study. Y. L., X. Z. and N. C. participated in study design and coordinated study centres. All authors contributed to the writing of the report. W. Y. is the guarantor of this article.

## Supporting information

Appendix S1
