# Supplementary material for: Determining the optimal fasting glucose target for patients with type 2 diabetes: Results of the multicentre, open‐label, randomized‐controlled FPG GOAL trial
Source: Diabetes Obes Metab. 2019 Jun 10;21(8):1973–7. doi: 10.1111/dom.13733 (PMC6772047; doi:10.1111/dom.13733)
Supplement: Supplementary file 1 — Appendix S1 [file DOM-21-1973-s001.docx]

# Supplementary methods

## Supplementary Table S1: insulin titration regimen.

| **FBG (mmol/L)** | **Insulin dose changes** |
| --- | --- |
| All groups <3.9 or nocturnal hypoglycaemia | −2 U |
| Group 2: 3.9<FBG≤5.6  Group 3: 3.9<FBG≤6.1 | Decrease 1–2 U or no change at investigator’s discretion |
| Group 1: 3.9<FBG≤5.6  Group 2: 5.6<FBG≤ 6.1  Group 3: 6.1<FBG≤7.0 | No change |
| Group 1: 5.6<FBG<10.0  Group 2: 6.1<FBG<10.0  Group 3: 7.0<FBG<10.0 | +2 U |
| All groups : FBG ≥10.0 | Increase 2–4 U at investigator’s discretion |
| FBG, fasting blood glucose; U, units | |

## Supplementary Table S2: hypoglycaemia categories.

| **Category** | **Definition** |
| --- | --- |
| Symptomatic | Symptoms of hypoglycaemia that respond to ingestion of carbohydrate or an episode associated with a blood glucose level of ≤3.9 mmol/L |
| Alert | A measured blood glucose level of ≤3.9 mmol/L with or without symptoms |
| Clinically important | A measured blood glucose level of ≤3.0 mmol/L with or without symptoms |
| Nocturnal | Hypoglycaemia occurring while the patient was asleep that either responded to ingestion of carbohydrate or was associated with blood glucose level ≤3.9 mmol/L |
| Severe | An event with clinical symptoms that are considered to result from hypoglycaemia in which the patient required the assistance of another person because the patient could not treat her/himself due to acute neurological impairment directly resulting from the hypoglycaemia, and one of the following criteria:  The event was associated with a measured blood glucose level <2 mmol/L  The event was associated with prompt recovery after oral carbohydrate, intravenous glucose, or glucagon administration |

## Statistical analysis

Mainly using data from the ATLAS treat-to-target study,[^1^](#_ENREF_1) in which the proportion of Chinese patients achieving an HbA1c <7% was 43.8% with an FPG target of 6.1 mmol/L,[^2^](#_ENREF_2) we calculated that 120 patients in Group 1 and 360 patients in Group 3 were required to achieve 85% power to detect a difference of 15% between groups (2-sided significant level of 0.05). Furthermore, to achieve 80% power to detect a difference of 10% between Group 2 and Group 3, 360 in each patient group was needed. Assuming a dropout rate of ~10%, the sample size for the total number of randomized patients was 934 (134 in Group 1, 400 in Group 2 and 400 in Group 3).

The primary and secondary endpoints were assessed in the full analysis set (FAS), defined as all randomly assigned patients who received at least one dose of study medication and had at least one post-baseline efficacy assessment. Safety was analysed in the safety population, defined as all randomly assigned patients who received at least one part or full dose of the study medication.

Pre-planned hierarchical testing was used for the primary endpoint: only if the difference between Group 1 and Group 3 was significant (nominal p-value <0.05), would testing proceed to compare the difference between Group 2 and Group 3. To compare between-group treatment effect differences, especially Group 3 as a control group, we further pre-specified exploratory multiple comparisons of interest using the Bonferroni correction method (adjusting p-value to 0.025). For all other endpoints, a nominal p-value <0.05 was used to determine statistical significance.

For the primary endpoint of patients achieving an HbA1c <7% and secondary endpoints of patients achieving an HbA1c <7% without hypoglycaemia, the crude proportions of patients achieving the goal at 24 weeks were calculated with associated 95% confidence intervals (CIs) estimated using the normal approximation to the binomial. For these analyses, missing data were imputed using the last observation carried forward (LOCF) method. Missing data were not imputed for the remaining secondary endpoints. The proportions of patients achieving an HbA1c <7% re-divided by actual FPG levels at 24 weeks were modelled using the same methods as above. Changes from baseline in HbA1c, FBG, PPG, insulin dose and bodyweight were modelled using ANCOVA with terms for treatment group and baseline value as covariate (Dunnett's test was applied for multiple comparisons between Groups 1 and 3 and Groups 2 and 3). Univariable linear regressions were performed to identify the numerical association between absolute HbA1c at 12 and 24 weeks and mean FBG during earlier (1–12 weeks) and later treatment (13–24 weeks), respectively, and multivariable linear regressions were also performed to assess these associations adjusted by baseline covariates including age, sex, BMI, diabetes duration, FPG, HbA1c, and PPG excursion (defined as the difference between self-monitored 2-hour PPG after breakfast and self-monitored FBG).

All statistical analyses were conducted using SAS (version 9.4).

# Supplementary results

## Supplementary Figure S1. Trial profile.

FBG, fasting blood glucose.

## Supplementary Table S3. Glycaemic outcomes and insulin dose at 24 weeks (full analysis set).

|  | **FBG Target, >3.9 mmol/L to** | | |
| --- | --- | --- | --- |
|  | **≤5.6 mmol/L (n=126)** | **≤6**.**1 mmol/L (n=393)** | **≤7**.**0 mmol/L (n=395)** |
| **HbA1c, %** |  |  |  |
| n | 119 | 367 | 377 |
| Mean ± SD | 7.06 ± 0.75 | 7.14 ± 0.92 | 7.30 ± 0.92 |
| LSM change ± SE | −1.49 ± 0.08 | −1.45 ± 0.04 | −1.28 ± 0.04 |
| P-value vs 3.9<FBG≤7.0 mmol/L | 0.033 | 0.010 |  |
| **HbA1c, mmol/mol** |  |  |  |
| n | 119 | 367 | 377 |
| Mean ± SD | 54 ± 8.2 | 55 ± 10.1 | 56 ± 10.1 |
| LSM change ± SE | −16.3 ± 0.9 | −15.8 ± 0.4 | −14.0 ± 0.4 |
| P-value vs 3.9<FBG≤7.0 mmol/L | 0.033 | 0.010 |  |
| **Self-monitored FBG,^†^ mmol/L** |  |  |  |
| n | 117 | 368 | 377 |
| Mean ± SD | 6.06 ± 1.07 | 6.42 ± 1.25 | 6.88 ± 1.37 |
| LSM change ± SE | −3.42 ± 0.12 | −3.11 ± 0.07 | −2.62 ± 0.07 |
| P-value vs 3.9<FBG≤7.0 mmol/L | <0.001 | <0.001 |  |
| **Serum FPG, mmol/L** |  |  |  |
| n | 119 | 367 | 377 |
| Mean ± SD | 6.62 ± 1.65 | 6.81 ± 1.65 | 7.27 ± 1.70 |
| LSM change ± SE | −3.91 ± 0.15 | −3.75 ± 0.09 | −3.28 ± 0.09 |
| P-value vs 3.9<FBG≤7.0 mmol/L | <0.001 | <0.001 |  |
| **PPG, mmol/L** |  |  |  |
| n | 106 | 313 | 327 |
| Mean ± SD | 10.45 ± 3.05 | 10.29 ± 2.89 | 10.69 ± 2.96 |
| LSM change ± SE | −3.33 ± 0.29 | −3.52 ± 0.17 | −3.16 ± 0.16 |
| P-value vs 3.9<FBG≤7.0 mmol/L | 0.842 | 0.224 |  |
| **Insulin dose, U** |  |  |  |
| n | 122 | 374 | 382 |
| Mean ± SD | 20.0 ± 11.1 | 19.5 ± 9.3 | 16.5 ± 8.5 |
| LSM change ± SE | 8.1 ± 0.7 | 6.8 ± 0.4 | 3.9 ± 0.4 |
| P-value vs 3.9<FBG≤7.0 mmol/L | <0.001 | <0.001 |  |
| **Insulin dose, U/kg.day**^‡^ |  |  |  |
| n | 126 | 393 | 395 |
| Mean ± SD | 0.28 ± 0.13 | 0.27 ± 0.12 | 0.23 ± 0.11 |
| **^†^**Defined as the mean value of the last three consecutive self-monitored FBG values.  ^‡^Global P-value assessing difference between the three FBG target groups <0.001 (ANOVA).  FBG, fasting blood glucose; FPG, fasting plasma glucose; LSM, least squares mean; PPG, postprandial glucose; SD, standard deviation; SE, standard error. | | | |

## Supplementary Table S4. Distribution of laboratory-measured fasting plasma glucose (FPG) and self-monitored fasting blood glucose (SM-FBG) at 24 weeks (full analysis set).

|  | **FBG Target, >3.9 mmol/L to** | | |
| --- | --- | --- | --- |
|  | **≤5.6 mmol/L (n=126)** | **≤6**.**1 mmol/L (n=393)** | **≤7**.**0 mmol/L (n=395)** |
| **Serum FPG (mmol/L)** |  |  |  |
| n | 119 | 367 | 377 |
| Distribution, n (%) |  |  |  |
| ≤5.6 | 38 (31.9) | 86 (23.4) | 55 (14.6) |
| 5.6–6.1 | 18 (15.1) | 59 (16.1) | 45 (11.9) |
| 6.1–7.0 | 20 (16.8) | 79 (21.5) | 80 (21.2) |
| >7.0 | 43 (36.1) | 143 (39.0) | 197 (52.3) |
| P value vs. 3.9<FBG≤7.0^a^ | <0.001 | <0.001 |  |
| **Mean SM-FBG (mmol/L)** |  |  |  |
| n | 117 | 368 | 377 |
| Distribution, n (%) |  |  |  |
| ≤5.6 | 47 (40.2) | 93 (25.3) | 50 (13.3) |
| 5.6–6.1 | 30 (25.6) | 74 (20.1) | 54 (14.3) |
| 6.1–7.0 | 22 (18.8) | 113 (30.7) | 132 (35.0) |
| >7.0 | 18 (15.4) | 88 (23.9) | 141 (37.4) |
| P value vs. 3.9<FBG≤7.0^a^ | <0.001 | <0.001 |  |
| **Minimum SM-FBG (mmol/L)** |  |  |  |
| n | 117 | 368 | 377 |
| Distribution, n (%) |  |  |  |
| ≤5.6 | 82 (70.1) | 176 (47.8) | 126 (33.4) |
| 5.6–6.1 | 17 (14.5) | 73 (19.8) | 68 (18.0) |
| 6.1–7.0 | 10 (8.5) | 81 (22.0) | 104 (27.6) |
| >7.0 | 8 (6.8) | 38 (10.3) | 79 (21.0) |
| P value vs. 3.9<FBG≤7.0^a^ | <0.001 | <0.001 |  |

^a^ Chi-squared test.

## Exploratory endpoints

When re-divided by actual 24-week FPG levels, the proportions of patients achieving an HbA1c<7% among those with a 24-week FPG ≤5.6mmol/L (68.7%, p<0.001) or >5.6 and ≤6.1 mmol/L (58.2%, p=0.013) were significantly greater than among those with a 24-week FPG >6.1 and ≤7.0mmol/L (43.6%). Furthermore, the proportion of patients achieving an HbA1c<7% among those with a 24-week FPG ≤5.6mmol/L was numerically, but not significantly, greater than among those with a 24-week FPG >5.6 and ≤6.1mmol/L (p=0.061).

Linear regression analysis demonstrated a significant association between lower FBG and lower HbA1c levels, which was consistently observed during both early (1–12 weeks; β coefficient = 0.371, p<0.001, R^2^ = 0.21, equation: HbA1c, % = 4.734 + FBG × 0.371) and later (13–24 weeks) treatment (β coefficient = 0.480, p<0.001, R^2^ = 0.23, equation: HbA1c, % = 4.055 + FBG × 0.480; Supplementary Figure S2). These associations remained after adjustment for baseline covariates including age, sex, BMI, diabetes duration, FPG, HbA1c, and PPG excursion (β coefficients = 0.348 and 0.498, respectively, both p<0.001).

## Supplementary Figure S2. Linear regression analyses for the association between mean self-monitored FBG and HbA1c during (A) early treatment (1–12 weeks, n=865) and (B) later treatment (13–24 weeks, n=863) (FAS).

FAS, full analysis set; FBG, fasting blood glucose. β coefficients are unadjusted.


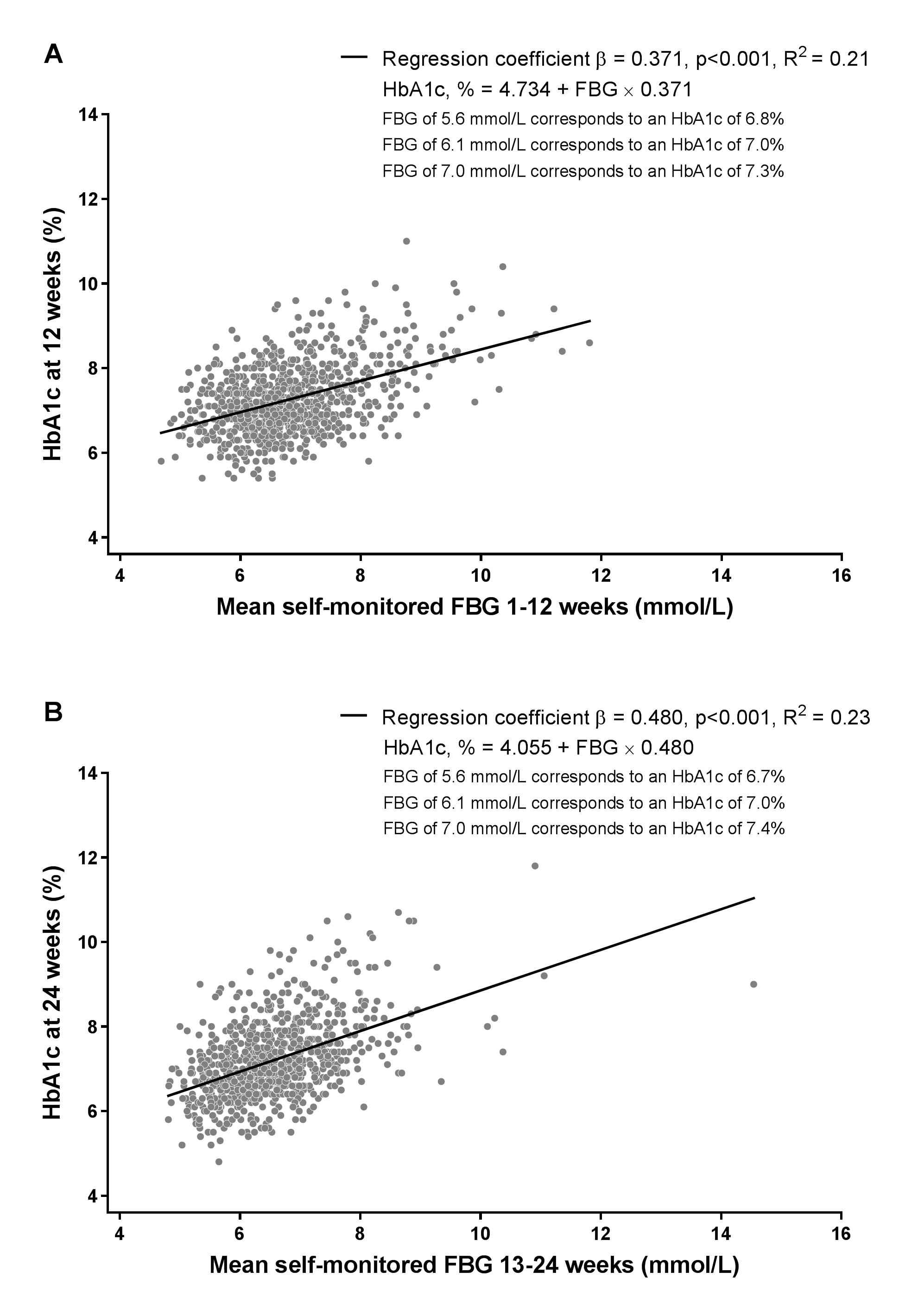


## Supplementary Table S5. Bodyweight and hypoglycaemia at 24 weeks (full analysis set), and other safety outcomes at 24 weeks (safety population).

|  | **FBG Target, >3.9 mmol/L to** | | | |
| --- | --- | --- | --- | --- |
|  | **≤5.6 mmol/L (n=126)** | **≤6**.**1 mmol/L (n=393)** | | **≤7**.**0 mmol/L (n=395)** |
| **Bodyweight, kg** |  |  | |  |
| n | 119 | 367 | | 378 |
| Mean ± SD | 70.0 ± 11.5 | 71.2 ± 12.1 | | 70.7 ± 11.4 |
| LSM change ± SE | 0.5 ± 0.3 | 0.7 ± 0.1 | | 0.7 ± 0.1 |
| P-value vs 3.9<FBG≤7.0 mmol/L | 0.745 | 0.988 | |  |
| **Hypoglycaemia**^†^ |  |  | |  |
| Any | 72 (57.1)*** | 169 (43.0) | | 145 (36.7) |
| Symptomatic | 53 (42.1)*** | 113 (28.8) | | 100 (25.3) |
| Alert (≤3.9 mmol/L) | 49 (38.9)*** | 108 (27.5) | | 92 (23.3) |
| Clinically important (≤3.0 mmol/L) | 6 (4.8) | 8 (2.0) | | 15 (3.8) |
| Severe | 0 | 1 (0.3) | | 1 (0.3) |
| Nocturnal | 25 (19.8)*** | 42 (10.7) | | 34 (8.6) |
| **Hypoglycaemia incidence, person-years (95% CI)**^†^ | | | | |
| Any | 3.6 (3.1–4.1)*** | 2.0 (1.8–2.2)** | | 1.6 (1.5–1.8) |
| Symptomatic | 1.9 (1.6–2.3)*** | 1.2 (1.1–1.4) | | 1.0 (0.9–1.2) |
| Alert (≤3.9 mmol/L) | 2.0 (1.7–2.4)*** | 1.1 (0.9–1.2) | | 0.9 (0.8–1.1) |
| Clinically important (≤3.0 mmol/L) | 0.1 (0.1–0.2) | 0.1 (0.0–0.1) | | 0.1 (0.1–0.1) |
| Severe | – | 0 (0–0) | | 0 (0–0) |
| Nocturnal | 0.8 (0.6–1.1)*** | 0.3 (0.3–0.4) | | 0.3 (0.2–0.4) |
| **Other safety outcomes, n (%)** | **(n=133)** | **(n=400)** | | **(n=403)** |
| Any AE | 91 (68.4) | 278 (69.5)^‡^ | | 243 (60.3) |
| Mild | 78 (58.6) | 219 (54.8) | | 197 (48.9) |
| Moderate | 13 (9.8) | 44 (11.0) | | 40 (9.9) |
| Severe | 0 | 14 (3.5) | | 6 (1.5) |
| Any AE except hypoglycaemia^§^ | 51 (38.3) | 188 (47.0) | | 168 (41.7) |
| Serious AEs | 4 (3.0) | 25 (6.3) | | 10 (2.5) |
| AEs leading to discontinuation | 0 | 7 (1.8) | | 1 (0.2) |
| AEs except hypoglycaemia occurring in ≥3% of patients^§^ | | |  | |
| Hyperlipidaemia | 1 (0.8) | 13 (3.3) | | 11 (2.7) |
| Nasopharyngitis | 7 (5.3) | 17 (4.3) | | 18 (4.5) |
| Toothache | 4 (3.0) | 3 (0.8) | | 4 (1.0) |
| URTI | 11 (8.3) | 44 (11.0) | | 37 (9.2) |
| ^†^Hypoglycaemia defined as per study criteria (Supplementary Table S2).  ^‡^One patient had an AE whose severity categorisation (mild/moderate/severe) was missing.  ^§^AEs including hypoglycaemia defined by MedDRA.  *p<0.05, **p<0.01, ***p<0.001 vs FBG ≤7.0 mmol/L group (nominal p-values). AEs, adverse events; CI, confidence interval; FBG, fasting blood glucose; LSM, least squares mean; SD, standard deviation; SE, standard error; URTI, upper respiratory tract infection. | | | | |

# Study investigators

Wenying Yang, China-Japan Friendship Hospital; Jinkui Ynag, Beijing Tongren Hospital, CMU; Hongmei Li, China Meitan General Hospital; Hui Pang, Tangshan Gongren Hospital; Ming Liu, Tianjin Medical University General Hospital; Heng Su, The First People’s Hospital of Yunnan Province; Yadong Sun, Jilin Province People’s Hospital; Qing Wang, Jilin University China Japan Union Hospital; Ling Li, Shengjin Hospital of China Medical University; Xulei Tang, The First Hospital of Lanzhou University; Minxiang Lei, Xiangya Hospital Central South University; Jianying Liu, The First Affiliated Hospital of Nanchang University; Xiaoyang Lai, The Second Affiliated Hospital of Nanchang University; Jingdong Liu, Jiangxi Provincial People’s Hospital; Jianyon Chen, The First People’s Hospital of Changde City; Weihong Song, Chenzhou NO.1 People’s Hospital; Xiaohong Lin, Zhuzhou Central Hospital; Yaoming Xue, Nanfang Hospital; Lishu Chen, The Second Affiliated Hospital of Shantou University Medical College; Daoxiong Chen, Hainan General Hospital; Xinjun Wang, The First Affiliated Hospital of Hainan Medical University; Xiaoyue Wang, The First People’s Hospital of Yueyang; Jianhua Ma, Nanjing First Hospital; Guoyue Yuan, Affiliated Hospital of Jiangsu University; Zhenwen Zhang, Northern Jiangsu People’s Hospital; Xinhua Ye, Changzhou No.2 People’s Hospital; Xiuzhen Zhang, Tongji University Affiliated Tongji Hospital Branch; Min Zhang, Qingpu Branch of Zhongshan Hospital; Wei Li, The Affiliated Hospital of Xuzhou Medical University; Chengjiang Li, The First Affiliated Hospital of Zhejiang University; Tianfeng Wu, Zhejiang Hospital; Jun Liu, The Fifth People’s Hospital of Shanghai, Fudan University; Riqiu Chen, Lishui City People’s Hospital; Haixiang Ni, Zhejiang Provincial Hospital of TCM; Heng Miu, The Second Affiliated Hospital of Nanjing Medical University; Qiuling Zhang, The Affiliated Hospital of Hangzhou Normal University; Zunhai Zhou, Yangpu Hospital, Tongji University; Changjiang Wang, The First Hospital of Anhui University; Tao Yang, Jiangsu Province Hospital; Shandong Ye, Anhui Provincial Hospital; Zhe Tang, Yan’an Hospital of Kunming City; Xiaolu Wang, Anshan Central Hospital; Qiaohua Ren, Shijiazhuang No.1 Hospital; Jun Wu, Wuhan Third Hospital.

# References

**1.** Garg SK, Admane K, Freemantle N, et al. Patient-led versus physician-led titration of insulin glargine in patients with uncontrolled type 2 diabetes: a randomized multinational ATLAS study. Endocr Pract. 2015;21:143-157.

**2.** Pan C, Tian H, Li Q, et al. Potential value of the Asian Treat to Target Lantus Study (ATLAS) for type 2 diabetes management in China: safety and efficacy of two treatment algorithms using insulin glargine. Chin J Endocrinol Metab. 2015;31:865-871.
